# Neuromodulation influences T lymphocyte calcium signaling and alpha synuclein clearance: implications for Parkinson’s disease

**DOI:** 10.3389/fncel.2025.1627305

**Published:** 2025-07-18

**Authors:** Alysia Ross, Natalie Prowse, Hui Zhang, Shawn Hayley, Hongyu Sun

**Affiliations:** ^1^Department of Neuroscience, Carleton University, Ottawa, ON, Canada; ^2^Department of Neurology, Queensway Carleton Hospital, Nepean, ON, Canada

**Keywords:** Parkinson’s disease, neuromodulation, T cell, alpha-synuclein, voltage-gated calcium channels

## Abstract

Along with the death of midbrain dopamine neurons, pathological accumulation of aggregated alpha synuclein (α-syn), often in the form of Lewy bodies, forms the hallmark pathological features of Parkinson’s disease (PD). Evidence of a neuroinflammatory response is a common secondary feature present in virtually all PD brains and is characterized by the presence of reactive microglial cells and signs of peripheral immune cell infiltration. Recent research has even suggested that PD might actually have an autoimmune component, as some PD patients express T cells that recognize epitopes specific to α-syn. Although neuromodulation is one of the leading treatment options for PD motor symptoms through regulating neuronal excitability, its impact on underlying α-syn and neuroimmune pathology remains poorly understood. While immune cells, such as T lymphocytes, have historically been thought to be non-excitable cells, their expression of L-type Ca^2+^ channels may suggest otherwise. In fact, these channels are thought to have an important role in CD4 + and CD8 + T cell proliferation, survival and cytokine release. Since emerging evidence suggests α-syn-specific T cell responses may be important in PD, we posit that neuromodulatory treatments may be useful for “reprograming” T cell functioning. In this review, we will summarize the role of α-syn specific T cell responses in PD pathology and consider the role of L-type Ca^2+^ channels on CD4 + and CD8 + T cells responses in the disease. We will then discuss the effect of neuromodulatory treatments on T cell-mediated immune responses and α-syn structure in an attempt to explain why treatments involving electrical stimulation are beneficial for PD patients.

## 1 Introduction

Parkinson’s disease (PD) is a neurodegenerative disorder that manifests clinically by tremor, bradykinesia, rigidity and gait disturbances along with many non-motor symptoms (Chaudhuri et al., 2006). It affects around 2% of individuals over the age of 65, and is about 2-fold more likely in men than in women. The exact etiology of PD remains poorly understood, but is most likely an interplay of several risk factors, including genetics and environment. Less than 10% of PD cases are strictly of genetic origin (mutations in PARK, SNCA, LRRK2 etc.) (Klein and Westenberger, 2012), with most being idiopathic in nature and likely involve environmental insults acting together with aging related changes in the brain. Regardless of the cause, excessive immune activation and resultant inflammatory processes have emerged as key players in PD onset and progression (Pajares et al., 2020; Caggiu et al., 2019; Weiss et al., 2022).

The hallmark features of PD are the degeneration of dopaminergic neurons in the substantia nigra pars compacta (SNc), along with accumulation of aberrant alpha-synuclein (α-syn), often in the form of Lewy bodies and Lewy neurites (Stefanis, 2012; Samii et al., 2004; Gómez-Benito et al., 2020). Although strictly genetic familial forms of PD are rare, there is a high probability that certain genes might act as vulnerability factors. In this regard, mutations in the SNCA gene, which is responsible for α-syn production, may be an important player in the inflammatory and neurodegenerative features of PD (Gómez-Benito et al., 2020). Hence, it is not surprising that high levels of aggregated α-syn are detected in post-mortem brain tissue of PD patients (Braak et al., 2003a). As well, α-syn aggregates are also found in the gastrointestinal (GI) tract many years before the onset of movement impairments (Zhu et al., 2022; Schaeffer et al., 2020; Braak et al., 2003b). In fact, GI impairments are an extremely common early biomarker of PD, and may be involved in disturbed gut-brain communication that occurs in the disease.

Much evidence has accumulated over several decades to indicate that neuroinflammatory processes are engaged in the PD brain (Caggiu et al., 2019; Lull and Block, 2010; Racette et al., 2018; Cebrián et al., 2015; Stolzenberg et al., 2017; Kannarkat et al., 2013). Regardless of the disease origins, reactive microglia are present in virtually all PD brains and infiltrating peripheral immune cells often accompany this neuroinflammatory feature (Lull and Block, 2010; Sanchez-Guajardo et al., 2010). In fact, systemic immune activation may play a critical role in PD, as elevated levels of CD4 + and CD8 + T cells have been reported both in the periphery and in the midbrain of PD patients (Galiano-Landeira et al., 2020). Such peripheral CD8 + T cells were reported to be able to migrate and penetrate the brain where they can directly contact dopaminergic neurons (Galiano-Landeira et al., 2020) and are correlated with neuronal death (Matheoud et al., 2016).

One important question that needs to be addressed is what particular signal could be activating peripheral immune cells, prompting their “homing in” on dopaminergic neurons? We posit that α-syn is the likely candidate for priming such an immune cell response. In support of this contention, direct overexpression of α-syn has been shown to promote infiltration of CD4 + and CD8 + T cells into the central nervous system (Olesen et al., 2018). Moreover, human postmortem studies have revealed the presence of T cells that recognize epitopes specific to α-syn in PD patient brains (Sulzer et al., 2017). Hence, it is possible that α-syn reactive T cells may contribute to dopaminergic cell death and eventual motor symptoms in PD through their direct actions on dopamine neurons, or possibly via secondary interactions with α-syn antigen presenting microglial cells.

In light of these findings, it is plausible that therapeutic interventions targeting immune mechanisms could influence the trajectory of PD. Voltage-gated calcium channels (VGCCs), particularly Cav1.2, are increasingly recognized as functional on CD4 + and CD8 + T cells, where they mediate calcium influx and downstream signaling. These pathways regulate T and B cell trafficking and cytokine production, which may in turn influence the spread and aggregation of α-syn. Additionally, α-syn itself contains charged domains that could respond to external electric fields. Thus, neuromodulatory treatments like ECT and TMS may exert dual effects—modulating T cell function through calcium signaling and potentially altering α-syn structure directly. These possibilities suggest a broader scope of action for neuromodulation in PD beyond neuronal circuit repair.

In this review we will discuss evidence that T cells may be involved in the α-syn aggregation and that the long-standing idea of these cells being “non-excitable” may not be entirely true. Recent evidence does indeed indicate that the activation, proliferation and migration of T cells depend, at least in part, on calcium (Ca^2+)^ release, which is maintained by voltage-gated calcium ion channels (VGCC) found on the T cell surface (Omilusik et al., 2011; Badou et al., 2013). Among these, the L-type calcium channel Cav1.2 has emerged as a key player in regulating calcium entry into CD4^+^ and CD8^+^ cells, influencing cytokine production and cell trafficking (Badou et al., 2013; Hurley et al., 2013; Branch et al., 2016). Accordingly, we currently postulate that neuromodulatory treatments that stimulate or block neuronal activation may also act through T-cell VGCCs, modulating their functions through calcium signaling pathways and that this might ultimately help to clear pathological α-syn.

In this review, we first examine the roll of T cells and T-cell mediated immunity in PD. We then consider how calcium signaling, particularly through VGCC, may impact α-syn and these immune processes. Next, we will summarize the evidence linking α-syn with immune system activation and its pathological role in PD. We will then discuss how neuromodulatory treatments can affect not only the activation of neurons but also T cells. Finally, and most importantly, we discuss how neuromodulation may be acting directly on VGCC on T cells that could provide critical insight into the use of neuromodulation as a treatment option for PD. We suggest that activation of Cav1.2 VGCC on CD4 + and CD8 + T cells can induce an influx of calcium resulting in downstream changes in Cav1.2 levels that influence T and B cell trafficking, which potentially modifies the aggregation of α-syn. Furthermore, we propose that neuromodulation may also act directly on α-syn itself, as opposing terminal charges (N^–^/C^+^) could, in theory, be susceptible to electric-field-induced conformational changes.

## 2 A brief immune system primer

The immune system is the primary defense mechanism against any environmental threats, ranging from microbial pathogens to neurotoxins and any other potential insults (Parkin and Cohen, 2001). The two broad divisions of immunity, the innate and adaptive, are crucial for rapid and long-term immune responses, respectively (Luckheeram et al., 2012). The innate branch which includes macrophages, neutrophils and brain microglia, responds rapidly to foreign threats and has the capacity to destroy pathogenic threats by phagocytosis (involving engulfment and degradation) or through the release of oxidative radicals and soluble inflammatory factors (Lull and Block, 2010). Although this is a very rapid immune response, the innate system is relatively non-specific and lacks true immunological memory. Dysregulation (particularly over-activation) of the innate immune response can result in excessive inflammation which is damaging to healthy tissue (Lull and Block, 2010; Kannarkat et al., 2013).

The adaptative immune response, which is often referred to as acquired or learned immunity, is a much slower, but highly specific process which has the capacity for long-term immune memory (Parkin and Cohen, 2001; Luckheeram et al., 2012). There are two main types of adaptive immunity: humoral and cellular. Humoral, or antibody-mediated immunity primarily offers protection against extracellular pathogens (especially bacteria), whereas cell-mediated immunity protects against intracellular pathogens (especially viral invaders). This system exists exclusively in mammals and higher organisms and consists of antigen-specific responses involving T (both CD4 + and CD8 +) and B lymphocytes. When a bacteria or toxin enters the extracellular space, B cells become primed and eventually produce antibodies which bind to these pathogens, neutralizing them through various mechanisms (Kannarkat et al., 2013; Parkin and Cohen, 2001). This is a two-step process that also requires the help of specific T cells, termed T helper cells (CD4 +), which provide an essential secondary signal. B cells can recognize intact antigens, whereas T cells recognize fragments of protein antigens that have already been partially degraded by antigen presenting cells, such as macrophages, neutrophils or even microglia (Alberts et al., 2002). These antigenic fragments are presented on the surface of antigen-presenting cells via major histocompatibility complex (MHC) molecules—MHC class II for CD4^+^ T cells and MHC class I for CD8^+^ T cells—allowing for specific immune activation. Intracellular infections prompt cytotoxic T cells (CD8 +), which are responsible for directly killing the infected cells. When dysregulation of such adaptive T and B cell-mediated immune responses occurs, autoimmunity can develop (Parkin and Cohen, 2001). In the case of PD, T cells have recently gained much attention as possibly contributors to the disease process, either directly or secondarily, in response to tissue damage.

### 2.1 T cell-mediated immune responses

#### 2.1.1 T helper cells (CD4 +)

**FIGURE 1:**
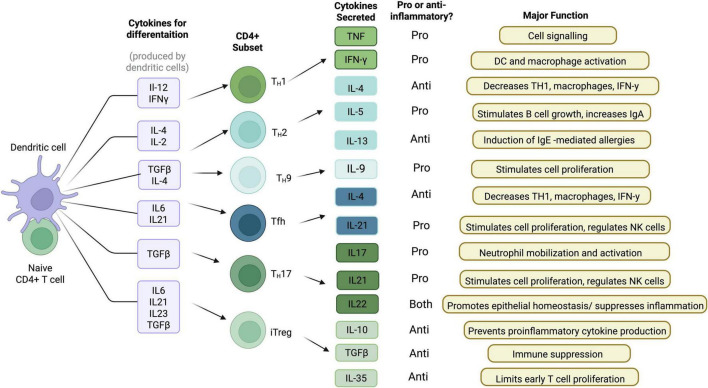
CD4 + T cell cytokine profile and function. When a naïve CD4 + T cell encounters a dendritic cell, it produces various cytokines for differentiation. Depending on the cytokines produced, specific CD4 + T cell subsets are developed. These different CD4 + T cell subsets produce various different cytokines that have proinflammatory functions, anti-inflammatory functions, or both (Created with BioRender.com).

Several types of CD4 + T helper cells have been identified, including Th1, Th2, Th17, as well as T regulatory cells. Overall, these cells are responsible for facilitating effective humoral or cell cytotoxic immune responses and this in part, depends upon specific cytokines (Figure 1). Indeed, T cells have distinct phenotypes depending on their cytokine profile. Th17 cells, characterized by their secretion of IL-17 and IL-22, are involved in pro-inflammatory responses and have been implicated in the pathogenesis of several neurodegenerative diseases, including PD (Bedoya et al., 2013). CD4 + Th1 and Th2 subtypes release IFN-γ, IL-2 and IL-4 (among others) which regulate B cell and CD8 + cytotoxic responses. In contrast, T regulatory (Treg) cells release the anti-inflammatory cytokine IL-10, to limit excessive pro-inflammatory signaling (Luckheeram et al., 2012).

#### 2.1.2 Cytotoxic T cells (CD8 +)

CD8 + cytotoxic T cells are responsible for killing cells that have an intracellular infection. Upon antigen recognition, CD8 + T cells become activated and produce TNF-a and IFN-y, which have potent anti-tumor and anti-microbial effects (Berg and Forman, 2006). These cells are extremely effective against viruses and are highly implicated in the immune response to HIV, hepatitis, Epstein Barr, influenza and other viruses than can impinge upon the brain (Shoukry et al., 2003; Jo et al., 2009; Sung et al., 2014; Riley and Montaner, 2017; Sung et al., 2015). Such effects come about through the activation of pro-apoptotic caspases or other pro-death pathways, aimed at killing the infected cell.

### 2.2 T cells in PD

Both CD4 + and CD8 + T cells have been found in PD post-mortem brain tissue (Brochard et al., 2009; Subbarayan et al., 2020), and their presence in both the CNS and periphery is now well supported (for a review, see Roodveldt et al., 2024). However, the timing of their appearance in the pathogenesis of PD is believed to vary. It is notable that CD8 + cell infiltration into the brain has been shown to precede the synucleinopathy that characterizes the early stages of PD (Galiano-Landeira et al., 2020). In contrast, CD4 + T cells may occur later in the progression of the disease (Weiss et al., 2022). Interestingly, CD8 + T cells were the dominant subtype observed to be associated with dopaminergic cell death in an MPTP toxin model of PD in rodents (Weiss et al., 2022; Brochard et al., 2009; Kurkowska-Jastrzebska et al., 1999b). Yet, further studies utilizing α-syn rodent models of PD reported that CD4 + were the primary T cell subtype associated with SN pathology (Harms et al., 2017).

Commonly used preclinical models of PD include the MPTP toxin model and α-syn-based models, each with distinct features. The MPTP model involves systemic administration of a neurotoxin that selectively targets and destroys dopaminergic neurons, leading to rapid degenerative changes and acute motor deficits (Smeyne et al., 2016). This model is widely used due to its reproducibility and strong resemblance to dopaminergic cell loss seen in PD. In contrast, α-syn models such as viral vector-based overexpression or injection of α-syn pre-formed fibrils aim to mimic the hallmark α-syn pathology and Lewy Body formation in PD, allowing researchers to investigate how α-syn aggregation and accumulation contribute to dopaminergic degeneration and immune system involvement. Given the differences in how pathology is induced, it is not surprising that MPTP and α-synuclein models exhibit distinct patterns of T cell involvement.

Beyond animal models, autoreactive T lymphocytes have been detectable in PD patient blood and CSF, as well as in animal models of the disease (Sulzer et al., 2017; Bandres-Ciga and Cookson, 2017; Bonam and Muller, 2020). Thus, there is some evidence that at least certain subtypes of PD have a degree of adaptive immune involvement, that can potentially act together with the innate neuroimmune component (which has already been supported by a much more substantial body of evidence). There remain open questions regarding exactly how the specific T cell subtypes might be involved in various aspects of PD. In particular, how might they modulate α-syn aggregation and how might the different T cells interact with resident microglia that are thought to control the general neuroinflammatory tone in PD?

Variations in several different T cells subtypes have been reported in the blood of PD patients, including decreased numbers of Treg cells, alongside increased numbers of CD4 + Th1 and Th17 cells (Chen et al., 2015; Chen et al., 2017). Furthermore, IL-17-producing CD4 + Th17 cells, isolated from PD patients, were reported to promote dopaminergic cell death (Bolte and Lukens, 2018). Similarly, CD4 + T cells have been linked to dopaminergic death observed in an MPTP rodent model of PD (Brochard et al., 2009). However, Treg cells were reported to be protective against MPTP-induced dopaminergic degeneration (Reynolds et al., 2007). In fact, copolymer-1 (Cop-1), a polypeptide that binds to MHC-II and is commonly used to treat autoimmunity in multiple sclerosis, stimulates Treg cells and elicits anti-inflammatory outcomes (Cruz et al., 2015). It could be that in PD patients, α-syn affects T cell homeostasis by early priming at peripheral sites (such as the gut) that ultimately causes an imbalance between T cell subtypes, favoring proliferation of CD4 + Th cells over the Treg subtype.

PD patients generally display symptoms of gut dysbiosis in the prodromal phase long before their primary diagnosis. In fact, constipation is a common early PD symptom that has been shown to worsen with disease progression and can precede motor symptoms by up to 10 years (Lin et al., 2014; Savica et al., 2009; Yu et al., 2018). Intriguingly, it has been suggested that the gut may be one location in which the adaptive immune system first becomes primed against α-syn aggregates (Garretti et al., 2019). This is supported by the finding that CD4 + T cells infiltrate the colon of PD patients who have constipation much more than those who do not (Chen et al., 2015; Sun et al., 2024). Furthermore, α-syn labeling has been observed in the colon in early stage PD, but is absent in healthy controls or in patients with irritable bowel syndrome (Shannon et al., 2012). Altered gut microbiota in PD patients is also associated with enteric α-syn pathology (Forsyth et al., 2011), though the nature of the relationship is unclear. It is however noteworthy that gut permeability, the impaired integrity of the intestinal lining that allows toxins to pass into the bloodsteam (Davies et al., 1996; van IJzendoorn and Derkinderen, 2019), and increased inflammation also commonly accompany such gastrointestinal disturbances (Unger et al., 2016). Forsyth and colleagues (Savica et al., 2009) were able to demonstrate a correlation between intestinal permeability and intestinal α-syn in newly diagnosed PD patients, suggesting that early stage α-syn pathology may underlie the intestinal symptoms, as well as possibly act as “seeds” for eventual brain pathology (Schmitt et al., 2023).

### 2.3 Role of microglia

Microglia are the brain’s resident innate immune cells, continuously remodeling their morphology and processes in order to optimally scan their microenvironment for any homeostatic alterations (Lull and Block, 2010; Schetters et al., 2018). In both *in vitro* and *in vivo* models, microglia internalize extracellular α-syn aggregates and attempt to degrade them (Weiss et al., 2022; Sanchez-Guajardo et al., 2010). However, this process can become impaired in PD, leading to the accumulation of pathogenic species (Lee et al., 2008). Chronic exposure to aggregated α-syn also leads to sustained microglial activation and a shift toward a pro-inflammatory phenotype, which not only reduces their phagocytic efficiency but also promotes the release of neurotoxic cytokines such as TNF-α, IL-1β, and reactive oxygen species (Boza-Serrano et al., 2014; Zhang et al., 2005).

Although microglia serve as the primary innate immune cells in the CNS, T lymphocytes represent their key peripheral adaptive immune system counterparts. Under normal physiological conditions, T cells are typically confined to the perivascular space and are thought to only appear sporadically in the healthy brain (Goverman, 2009; Cebrián et al., 2014). However, recent studies using advanced imaging and immunological analysis have demonstrated that tissue-resident memory T cells (including both CD4 + and CD8 +) can persist within the brain parenchyma and contribute to immune surveillance and CNS homeostasis (Smolders et al., 2018). Additionally, gut-derived T cells can migrate to specific brain regions under steady-state conditions in both mice and humans (Yoshida et al., 2025). These findings highlight a more active, dynamic role for peripheral adaptive immune responses in maintaining brain health and suggest that T cell-microglia cross-talk in physiological conditions may be more important than previously thought. While the individual roles of microglia and T cells in PD have already been extensively reviewed (Schetters et al., 2018), much less attention has focused on T cell interactions with microglia in this regard. This highlights the need for further research into how microglia–T cell signaling may influence α-syn clearance and dopaminergic survival in PD.

α-Syn pathology may also modulate microglia–T cell interactions through MHC signaling pathways. For example, exposing microglia to α-syn caused dopaminergic neurons to express the major histocompatibility complex (MHC) molecule (Sulzer et al., 2017; Cebrián et al., 2014). Normally, MHC I and MHC II enable antigen presentation to CD8 + and CD4 + , respectively. While MHC I are expressed on almost all nucleated cells, MHC II are only expressed on certain antigen presenting cells (APC), including microglia. In genome wide association studies, PD has been associated with an immune haplotype that includes specific MHC II alleles (Hamza et al., 2010; Kannarkat et al., 2015). Furthermore, α-syn overexpression models have reported increased MHC II expression (Subbarayan et al., 2020) and other animal models of PD reported that infiltrating CD4 + T cells were surrounded by MHC-II expressing microglia, supporting the notion of innate and adaptive immunity cross-talk (Subbarayan et al., 2020; Earls et al., 2019; Sanchez-Guajardo et al., 2013). The role of MHC-II in dopaminergic neurodegeneration was further strengthened by the observation that MHC-II null mice failed to show dopaminergic pathology in an α-syn overexpression model (Harms et al., 2013).

## 3 Calcium signaling and the immune response

**FIGURE 2:**
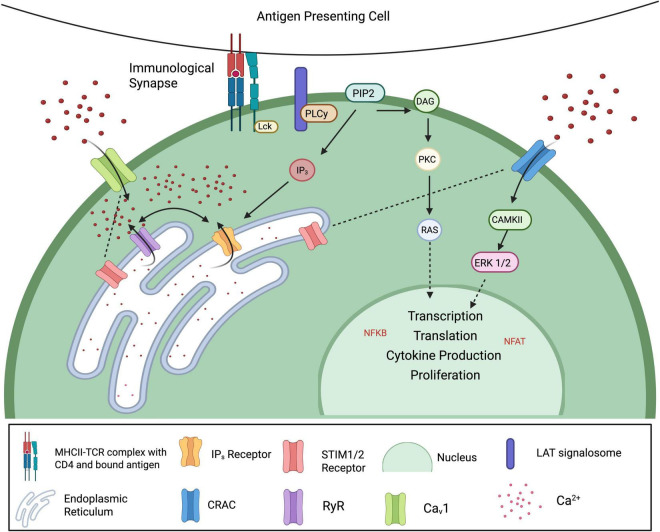
Calcium signaling in T lymphocytes. Antigen recognition by T cells activates the T cell receptor (TCR), triggering phosphorylation of cytoplasmic ITAMs by Src family kinases such as Lck and Fyn. This leads to the recruitment and activation of ZAP-70, which phosphorylates LAT. Binding of PLCγ to phosphorylated LAT catalyzes the hydrolysis of PIP_2_ into two key second messengers: IP_3_ and DAG. IP_3_ stimulates calcium release from the endoplasmic reticulum (ER), while DAG activates PKC and Ras, ultimately leading to the activation of transcription factors such as NF-κB and NFAT. As ER calcium stores deplete, conformational changes in STIM1/2 allow them to translocate to the plasma membrane and activate CRAC and CaV1 channels, permitting sustained Ca^2+^ influx. This elevation in intracellular calcium drives the activation of downstream effectors, promoting transcription factor activity, cytokine production, and T cell proliferation (Created with BioRender.com).

An effective lymphocyte immune response requires intracellular signaling molecules that are responsive to stimulation with rapid time-dependent variations in concentration (Trebak and Kinet, 2019; Vig and Kinet, 2009). It is well established that Ca^2+^ is a second messenger in many types of cells, including being crucial for T cell activation, proliferation and function (Trebak and Kinet, 2019; Vaeth et al., 2020). The normal chain of events that occurs when an APC activates the membrane-bound T cell receptor (TCR), is the phosphorylation of several intracellular kinases, including the activation of immunoreceptor tyrosine-based activation motifs (ITAMs). This cascade leads to the release of two critical second messengers: inositol trisphosphate (IP3), which triggers calcium release from the endoplasmic reticulum, and diacylglycerol (DAG), which activates protein kinase C (PKC) and contributes to downstream transcriptional responses (Kummerow et al., 2009; Figure 2). When T cells subsequently re-encounter a known antigen, their Ca^2+^ response is rapidly augmented, resulting in a type of long-term immune memory.

### 3.1 The immunological synapse

During an adaptive immune response, APCs are responsible for the initial processing of foreign antigens into peptides that are packaged into MHC molecules. At the same time, T cells are constantly patrolling their surroundings looking for antigen-MHC complexes that match their specific TCRs. The molecular interaction between T cells and MHC complexes is referred to as the immunological synapse (IS) (Vaeth et al., 2020; Kummerow et al., 2009). The interaction of the antigen-specific TCR-MHC at the IS causes greatly increased intracellular Ca^2+^ cytosolic levels (Trebak and Kinet, 2019; Vaeth et al., 2020; Kummerow et al., 2009). Ca^2+^ is important for the formation of a mature IS and induces cell polarization, involving the re-distribution of cytosolic signaling proteins and re-localization of organelles (Sánchez-Madrid and del Pozo, 1999). Any particular IS can exist from a few seconds to up to several hours and results in both local and global Ca^2+^ signaling in T cells that is crucial for their activation. A short IS duration is thought to be beneficial for naïve CD4 + and CD8 + T cells that are scanning their environment for matching APCs, while longer IS durations are more typical during cytotoxic CD8 + T lymphocyte-dependant cell killing (Kummerow et al., 2009; Stinchcombe et al., 2006). The IS is also known to be very different between CD4 + and CD8 + T cells and their respective MHC molecules on APCs. CD8 + T cells are thought to form stable synapses, that can maintain contact with the same APC over hours. On the other hand, CD4 + T cells form something called a kinapse, which only maintains communication for a few minutes (Dustin, 2009). While these mechanisms remain incompletely understood, Ca^2+^ signaling is clearly critical for both the formation and maturation of the IS and the functional outcome of T cell-APC interactions.

### 3.2 Calcium channels in T cells

Calcium signaling in T cells is important for virtually all aspects of T cell function, including activation, proliferation, differentiation and effector responses. Upon T cell receptor (TCR) engagement, calcium influx from the extracellular space and calcium release from intracellular stores act together to drive critical downstream signaling pathways (Figure 2). This is achieved through a diverse array of calcium-permeable channels, including calcium-release activated channels (CRAC), voltage gated calcium channels (VGCC) and members of TRP and P2X (Trebak and Kinet, 2019). The diversity of calcium entry mechanisms ensures that T cells can easily fine tune their responses to varying immune challenges.

#### 3.2.1 Calcium-release activated channels

The most studied T cell membrane channels are the calcium release-activated channels (CRAC), which are formed by a combination of calcium release-activated calcium channel protein (ORAI) plasma membrane proteins (ORAI1, ORAI2, ORAI3). Following IP_3_ receptor opening, endoplasmic reticulum Ca^2+^ levels decrease, causing a conformational change in stromal interaction molecules 1 and 2 (STIM1, STIM2), which act as ER calcium sensors. Upon calcium depletion, STIM proteins translocate to the plasma membrane, where they interact with and open ORAI channels (ORAI1-3), facilitating sustained Ca^2+^ entry. The resulting Ca^2+^ influx triggers various intracellular processes, including the activation of Ca^2+^ dependent enzyme CAMKII and Erk1/2 and transcription factors NFκB and NFAT (Vaeth et al., 2020; Figure 2).

The importance of ORAI channels in calcium-mediated intracellular responses has been demonstrated in mouse models. Indeed, ORAI1 knockout mice show decreased store-operated Ca^2+^ entry (SOCE) and ORAI1/ORAI2 double-knockouts exhibited completely abolished SOCE and impaired T cell function (Vaeth et al., 2017; Xing et al., 2014). Furthermore, loss of function mutations in STIM1 and ORAI1 had completely abolished CRAC channel function that was associated with severe combined immunodeficiency (SCID)-like disease and autoimmunity (Lacruz and Feske, 2015). SOCE-deficient T cells show resistance to apoptotic cell death and their ability to recall antigens in both human patients and mice is severely impaired (Feske et al., 1996; McCarl et al., 2010). In neurons, loss of SOCE has been shown to trigger ER stress and the degeneration of dopaminergic neurons in a manner that is reflective of what happens in PD (Stefani et al., 2012), though it is unclear what role SOCE-deficient T cells might play in the disease.

#### 3.2.2 L-type voltage-gated calcium channels

Other calcium channels found on T cells include the L-type calcium channels. These voltage gated Ca^2+^ channels normally play an important role in the function of electrically excitable cells, such as neurons following depolarization. However, several studies have shown that T cells also express high levels of the Ca_*v*_1 pore-forming subunit subfamily (Ca_*v*_1.1-1.4) (Badou et al., 2013; Badou et al., 2006; Badou et al., 1997) But their role in Ca^2+^ influx and ability of T cells to be depolarized and evoke inward Ca^2+^ remains controversial. Initially, the inhibitory effects of L-type Ca_*v*_ antagonists suggested that human T cells express functional Ca_*v*_ channels (Stokes et al., 2004). However, at high doses these drugs also block K + channels which can inhibit calcium signaling via CRAC channel depolarization (Stokes et al., 2004; Hogan et al., 2010; DeCoursey et al., 1985). While it has been suggested that Ca_*v*_1 channels can be activated by either TCR stimulation or depolarization, this could result in inappropriate immune responses should voltage-dependant opening occur in the absence of an appropriate TCR stimulus (Badou et al., 2013). However, if there is already an antigen-specific T cell response ongoing, then voltage stimulation independent of TCR could conceivably strengthen the existing immune response and cause greater antigen clearance. Ca_*v*_ channel activation and subsequent Ca^2+^ changes may also conceivably occur through some unknown biochemical pathway, rather than strictly from membrane depolarization *per se*.

### 3.3 Calcium signaling and T cell activation

It is already known that CD4 + and CD8 + T cells express high levels of the Ca_*v*_1 subunit family (Ca_*v*_ 1.1-1.4) and that Ca_*v*_1.1 expression is upregulated through antigen-TCR activation. Moreover, Ca_*v*_1.2 and 1.3 are expressed on effector Th_2_ CD4 + T cells, whereas Ca_*v*_1.4 is expressed on both naïve CD4 + and CD8 + T cells. The role of these channels became apparent following the observation that a mutation in the β4 subunit gene, responsible for normal Ca_*v*_ function, results in immunological disorders and a deficit in cell-mediated immune responses in mice (Morrison et al., 1984). Furthermore, CD4 + T cells lacking either β3 or β4 have impaired SOCE, NFAT activation and cytokine production (Badou et al., 2006). The impaired Ca^2+^ influx in β3 deficient mice is thought to be directly associated with a lack of Ca_*v*_1.4 protein expression (Omilusik et al., 2011; Jha et al., 2009).

To our knowledge, no studies have investigated L-type Ca^2+^ channels in relation to T cell peripheral activation and migration into the brain. It is generally accepted that these channels only become relevant following T cell activation and that this occurs outside the brain (Goverman, 2009). Supporting this idea, Kv1.3—a voltage-gated potassium channel upregulated in effector memory T cells—has been implicated in regulating T cell activation, proliferation, and migration. Notably, it has been demonstrated that there is little to no Kv1.3 expression in T cells in the grey or white matter of healthy brain tissue. However, brains from multiple sclerosis patients (which involves autoimmune T cell recruitment) show high levels of Kv1.3 positive CD4 + T cells (Rus et al., 2005). This is consistent with the possibility that functional ion channels in T lymphocytes undergo development and activation in the periphery before being recruited to the brain. The implication for PD being that antigen-specific responses to α-syn may begin prior to brain infiltration.

## 4 α-syn: form and function

α-syn is a highly soluble protein composed of 140 amino acid residues that is encoded by the SNCA gene. The central, or “non-amyloid component” (NAC) region, permits the oligomerization of α-syn due to its hydrophobic composition (Ulmer et al., 2005; Bernal-Conde et al., 2020) and the N-terminal region contains imperfect repeats allowing the formation of an alpha-helix through lipid-binding (Bartels et al., 2010; Segrest et al., 1990). α-syn also contains two oppositely charged structural domains that are crucial for α-syn folding: the positively charged N-terminal lipid-binding domain, and the negatively charged C-terminal domain (McClendon et al., 2009; Ross et al., 2020). Thus, electrical current–based approaches, as described in this paper, may offer a means of targeting the α-synuclein molecule directly, independent of cellular mechanisms.

Normally, α-syn exists as a presynaptic amyloid protein that is a major constituent in intracellular pathological Lewy aggregates. While many environmental and genetic factors can influence α-syn aggregation, the exact triggers and/or facilitators are not fully understood. In PD, α-syn pathology is found both within the soma and dendritic processes in various brain regions (Stefanis, 2012; Brás and Outeiro, 2021): distribution varies at different stages of the disease and theories regarding its initial location and progression toward Lewy neurites have been proposed. Braak et al. (2003a) have studied post-mortem PD brains and tissues resulting in their postulation that in some cases of PD the α-syn pathology begins in the GI tract and then travels to the brainstem through the vagus nerve, gradually following an ascending course progressively forward in the brain. However, this hypothesis does not clearly define why, exactly where, and how α-syn aggregation begins.

### 4.1 Monomers

Although the understanding of exact physiological function of α-syn monomers in the healthy brain remains incomplete, the protein is believed to play an important role in synaptic function and in particular, presynaptic vesicle trafficking. Indeed, overexpression of α-syn has been shown to induce a decrease in readily releasable vesicles, especially in dopaminergic neurons (Bridi and Hirth, 2018). SNCA knock-out also causes an impairment in synaptic responses to prolonged high frequency stimulation of hippocampal neurons (Cabin et al., 2002). The release of neurotransmitters, including dopamine, is regulated by the pre-synaptic SNARE protein complex and the assembly, maintenance and degradation of this protein has been shown to be controlled by α-syn (Bridi and Hirth, 2018; Burré, 2015). Together, these results suggest that α-syn plays an important role in vesicle trafficking, docking and release. Furthermore, a neuroprotective role of the monomer form of α-syn has been suggested following the observation that it can inhibit apoptosis in response to various pro-apoptotic stimuli (Alves Da Costa et al., 2002). However, it is important to the note that α-syn knockout mice are still viable, suggesting the physiological role of α-syn is non-essential for basic synaptic machinery. Rather than being strictly required, α-syn may play a modulatory role in synaptic plasticity and stress responses that becomes more apparent during aging, where its absence may contribute to vulnerability under specific pathological conditions (Greten-Harrison et al., 2010). This underscores the complexity of α-syn biology—where both its loss and its aggregation may be detrimental depending on context.

### 4.2 From monomer to fibril

Monomeric α-syn is an intrinsic, soluble protein found both in the GI tract and the brain and has been reported to possess anti-viral properties. In response to viral infections, α-syn is expressed in GI enteric neurons and α-syn knockout increased susceptibility to viral encephalitis (Massey and Beckham, 2016). Further, levels of α-syn in the gut of children with duodenal inflammation correlate positively with the degree of immune cell infiltration (Stolzenberg et al., 2017). *In vitro* models have also demonstrated that monomeric α-syn can act as a chemoattractant for neutrophils and monocytes, as well as a maturation factor for dendritic cells (Stolzenberg et al., 2017).

The aggregation of α-syn monomers results in the generation of oligomers, and mounting evidence suggests they are neurotoxic (Rodriguez et al., 2018; El-Agnaf et al., 2006; Yamamoto et al., 2019). Besides their extracellular aggregation, α-syn monomers can accumulate together within the mitochondria to form toxic oligomers that cause ER stress, mitochondrial dysfunction, cell apoptosis and inflammation (Colla et al., 2012; Chinta et al., 2010; Lee et al., 2010; Deas et al., 2016). This cytotoxicity has been related to changes in membrane potential and the loss of calcium homeostasis (Yamamoto et al., 2019; Luth et al., 2014). In addition, the A53T α-syn mutation further exacerbates mitochondrial damage in conjunction with α-syn aggregates (Chinta et al., 2010). Furthermore, a positive feedback loop between α-syn oligomers and ER stress exists, whereby ER stress can increase the level of α-syn oligomers, which in turn increase ER dysfunction.

It is reasonable to assume that native α-syn exists predominantly as a monomer, though it clearly can form an oligomer or fibril under specific stress-induced conditions. *In vitro* and *in vivo* models show that specific point mutations in the SNCA gene (i.e., E46K and A53T) can accelerate α-syn oligomerization and enhance fibrilization (El-Agnaf et al., 2006; Conway et al., 1998; Conway et al., 2000). The conversion of α-syn to a toxic fibrillar form is thought to be due lipid interaction and phosphorylation of α-syn at Ser129 and Ser87 (Oueslati, 2016; Oueslati et al., 2012). Remarkably, oligomeric α-syn phosphorylated at Ser129 (pSer129), obtained from the brains of dementia with Lewy body (DLB) patients, has been shown to directly convert recombinant monomers into fibrils (Sano et al., 2018), suggesting once again that this protein has prion-like capabilities. In fact, numerous studies have shown that α-syn can spread from cell to cell in a prion-like fashion, triggering the formation of α-syn fibrils and Lewy bodies (Mao et al., 2016). Importantly, most studies that have investigated the seeding and conversion of α-syn to oligomers and fibrils have focused on brain tissue, so it is still unclear whether these processes unfold in exactly the same manner in the other tissues, including the GI tract. This is a vital question since α-syn aggregates can ascend the vagal nerve in response to pathological stimuli in PD animal models (Kim et al., 2019; Wood, 2019; Holmqvist et al., 2014).

### 4.3 α-syn and T cells

An adaptive response requires some specific antigenic stimulus and in the case of PD, emerging data suggest that α-syn might serve such a role. Indeed, the fact that CD4 + T cells of PD patients were found to recognize α-syn peptides has fueled the idea of T cell responses directed against α-syn (Sulzer et al., 2017). Moreover, it has been suggested that CD8 + T cell infiltration and interaction with MHC I on SNc dopaminergic neurons may occur in certain cases of PD (Sulzer et al., 2017). Whether CD8 + T cell infiltration and possible interactions with neuronal MHCI are a cause of neurodegeneration or simply a reaction to damaged DA cells is controversial. It is noteworthy that the loss of CD8 + T cell activity in CD8 + knockout mice did not provide protection of dopaminergic neurons (Schetters et al., 2018; Gundersen, 2021; Williams et al., 2021), but genetic deletion of CD4 reduced brain MHCII responses to α-syn (Williams et al., 2021).

If CD4 + T cells can recognize α-syn-specific epitopes, then they could participate in the clearance of toxic α-syn. This suggests that some immune responses are beneficial in PD, which remains controversial, as most studies report that α-syn-induced inflammatory responses contribute to PD pathology (Caggiu et al., 2019; Stolzenberg et al., 2017; Harms et al., 2013; Bartels et al., 2010). While evidence exists that CD4 + and CD8 + T cells contribute to dopaminergic neuron loss in PD (Galiano-Landeira et al., 2020; Brochard et al., 2009; Subbarayan et al., 2020; Kurkowska-Jastrzebska et al., 1999b), other studies suggest that specific T cell subsets, such as Tregs, may exert neuroprotective effects (Reynolds et al., 2007). This duality indicates that the impact of T cells in PD is highly context-dependent. Our hypothesis that neuromodulation may beneficially “reprogram” T cell function is speculative and should be approached with caution, as excessive immune activation may exacerbate neurodegeneration. It is also possible that T cells may clear toxic α-syn independent of or before any cell death occurs, since CD4 + T cell increases were observed in some α-syn overexpression models in the absence of any dopaminergic cell death (Sanchez-Guajardo et al., 2010). In this case, one could imagine that immunosurveillance is acting in a prophylactic manner in an attempt to restrain any α-syn toxicity. There may be a “sweet spot” in modulating T cells, that could have beneficial effects preventing α-syn-induced PD progression while not being aggressive enough to cause any “bystander” damage to otherwise healthy neurons. Indeed, any possible beneficial effects of T cell mediated a-syn clearance should be balanced against the possibility that overzealous autoimmunity could potentially contribute to dopaminergic degeneration (Bonam and Muller, 2020; Chang et al., 2018). It has been speculated that in PD, some T cells may have escaped tolerance during thymic selection (Marrack and Kappler, 2012), or alternatively, that intracellular α-syn might cause distressed neurons to produce novel antigens or “neoepitopes” that could be targeted.

It could be that T cell targeting of extracellular α-syn might be protective by helping clear the protein before entering the cell, whereas targeting neurons containing intracellular Lewy inclusions would presumably increase the death of such neurons. Regardless, the idea of T cell modulation for α-syn clearance is quite new and likely involves two specific antigenic regions of the α-syn protein that can elicit either MHC I or MHC II responses. The first is the Y39 N terminus region and (Sulzer et al., 2017), whereas the second antigenic region near the s129 C terminus (Sulzer et al., 2017). The majority of cell responses to the α-syn peptides produced the cytokine, IL-5, and comprised a CD4 + Th2/Class II T cell response (68%, versus 32% IFNγ CD8 + /Class I responses) (Sulzer et al., 2017). This suggests that T cell responses to α-syn may be primarily mediated by IL-5 secreting CD4 + T cells which, as previously mentioned, are directly regulated by Ca^2+^ responses.

## 5 Neuromodulatory treatments

The effects of neuromodulatory treatments on T cells has been relatively unexplored, despite evidence that they may modulate microglial responses (Svensson et al., 2021; van Buel et al., 2015). Yet, neuromodulatory treatments are a popular treatment option for PD. Deep brain stimulation, introduced in 1987, is a well-established treatment for motor impairment in advanced staged PD, whereas less-invasive techniques such as electroconvulsive therapy (ECT), transcranial direct current stimulation (tDCS) and transcranial magnetic stimulation (TMS) are more commonly used to treat co-morbid depression that is often evident in PD patients (Manenti et al., 2018; Ganguly et al., 2020). While TMS has been used on occasion to treat the motor symptoms of PD patients, the results are mixed, likely due to the fact that TMS can only target cortical regions and does not directly reach the basal ganglia (Fregni et al., 2005). In this discussion, we will focus on the use of ECT and tDCS/TMS as they are non-invasive and we believe have the potential to modulate a-syn clearance, possibly by influencing T cell and microglia interactions.

### 5.1 Deep brain stimulation

Deep brain stimulation is the most performed surgical procedure for advanced stage PD. It involves the implantation of electrodes into the subthalamic nucleus (STN), or caudal zona incerta (cZI) and the delivery of electrical signals via a battery source (Martinez-Ramirez et al., 2015; Musacchio et al., 2017; Fang and Tolleson, 2017; Table 1). Its advantages include focality and reliable improvements of motor symptoms, especially tremor. Furthermore, the efficacy of this treatment appears to depend upon stage of disease (and hence, the number of neurons that are still viable), since STN-DBS benefits are greatest in early stages of the disease (Merola et al., 2015). DBS is believed to work by acting on neurons and adjacent fiber tracts in the targeting region, resulting in neurotransmitter release and modulation of neuronal circuits. STN-DBS suppresses activity of GPi neurons resulting in marked improvements in motor symptoms (Chiken and Nambu, 2014). However, the exact mechanisms behind the treatment effects of DBS remain unknown.

**TABLE 1:** Comparing the parameters of DBS, ECT and tDCS as it relates to the treatment of PD.

Parameter	DBS	ECT	tDCS
Pulse width:	Microseconds (μs) average: 82 μs	Milliseconds (ms) or seconds (s) average: 0.25–1 ms	N/A
Amperage	2–8 mA	0.9 A (constant current)	1–2 mA (Anodal)
Voltage	2.4–3 V	70–120 V	9 V battery
Frequency	136–152 Hz	50–70 Hz	0.75–5 Hz
Target	STN, GPi	Depends on electrode placement. widespread	motor cortex, DLPFC
Number and duration of sessions	N/A (continuous)	1–3/week for total of 6–12 sessions. 7–14 s per session	20–30 min (anodal stimulation) for 2–3 sessions
Duration of effect	Seconds	7 days +	1–2 h
Induced EEG seizure	N/A	30–60 s	N/A

Relevant to this discussion, studies have reported that DBS-STN influences immune cell activity including increasing natural killer cells, IL-6 and TNF-α and decreasing B cell numbers (Grembecka et al., 2021; McFleder et al., 2025). However, these effects are often observed in the periphery and do not necessarily reflect the changes occurring in the brain. More recent work has demonstrated that DBS may act on astrocytes, leading to increased Ca^2+^, glutamate release, and subsequent postsynaptic slow inward currents in neighboring neurons which promotes synchrony of neuronal firing (Fenoy et al., 2014; Hamilton and Attwell, 2010). Furthermore, In both human patients and the A53T α-synuclein rat model, DBS was shown to suppress pro-inflammatory Th17 responses, enhance regulatory T cells, and reduce neuroinflammation, supporting a potential disease-modifying role through immune modulation (McFleder et al., 2025). Despite T cells displaying similar calcium dynamics to astrocytes, no known studies have investigated the effects of DBS on T cells in the brains of PD patients. Immunomodulatory effects of DBS on T cells and long-term immune changes warrant further exploration.

### 5.2 Electroconvulsive therapy

Clinically, ECT has been a popular psychiatric treatment option for severe depression and its efficacy requires the induction of generalized seizures (Popeo and Kellner, 2009). A pre-defined voltage is applied to the brain, resulting in a direct current passing between the electrodes from temple to temple (bilateral) or front to back (unilateral) (see Table 1 for summary of effects). The electrical impulses from ECT result in the stimulation of neurons via alternations in their internal electrical milieu and ionic concentrations (Swartz, 2014; Singh and Kar, 2017). Acute ECT results in upregulation of various immune cells, whereas repetitive ECT results in the suppression of immune responses (van Buel et al., 2015; Singh and Kar, 2017; Guloksuz et al., 2014; Di Gennaro et al., 2016; Fischler et al., 1992). ECT has also been shown to enhance responsive motility and purinergic currents in mouse microglia, as well as promote neurogenesis in the adult hippocampus (Sepulveda-Rodriguez et al., 2019). Given these findings, it has been suggested that ECT-induced modulation of inflammatory responses may have therapeutic benefits (van Buel et al., 2015).

Although ECT is currently only used as a treatment option for depressive symptoms in various psychiatric disorders, several studies indicate it can reduce motor symptoms in PD patients (Popeo and Kellner, 2009; Grover et al., 2018; Fall et al., 1995; Usui et al., 2011; Rodin et al., 2021). In a PD patient who was unresponsive to L-Dopa, eight sessions of bilateral ECT twice a week resulted in significant improvements in the number of steps taken in 7 m and freezing episodes (Pintor et al., 2012). Furthermore, a case study of a 74 year old female with tardive tremor resulting from dopamine blocking agents saw significant improvement in both depressive symptoms and tremor following 10 ECT sessions (Yeh et al., 2020). Relative to more invasive neuromodulatory treatments such as DBS, the application of ECT is much safer, cheaper and has been supported as a more common treatment option for treating both cognitive and motor symptoms of PD (Kellner and Kellner, 2015).

### 5.3 Transcranial direct current stimulation (tDCS and rTMS)

Like ECT, tDCS uses electrodes on the scalp to apply an electric current to the brain. Depending on the polarity of the current, it can be used to increase or decrease neuronal firing. The differences between the procedures are related to differences in parameters, as outlined in Table 1. Stimulation with tDCS mainly targets the motor cortex and dorsolateral prefrontal cortex (DLPFC), which are directly related to the motor and cognitive symptoms of PD, respectively. Strikingly, tDCS has been shown to improve gait and cognitive impairment following stimulation of both the motor cortex and DLPFC, but not the motor cortex alone (Dagan et al., 2018). However, other studies with only motor cortex stimulation electrode placement have reported beneficial effects on various motor symptoms of PD (for a review, see Ferrucci et al., 2016). Notably, Luo et al. (2022) have shown that the closely related neurostimulation procedure, rTMS, inhibited NFκB, which is involved in transcriptional regulation of cytokine and chemokine expression, and others have reported that rTMS reduced the release of proinflammatory cytokines and inhibited microglia activation (Yang et al., 2020; Zong et al., 2020; Kim et al., 2013). More recently, it has been demonstrated that a 10-day course of 10Hz rTMS over the motor cortex alleviated motor dysfunction in PD patients and MPTP-treated mice, effects that were correlated with increased peripheral regulatory T cells, suppressed microglial activation, decreased proinflammatory cytokines, and implicated Syt6 as a key mediator (Xie et al., 2024).

While DBS, ECT, tDCS and rTMS are all capable of modulating neuronal activity, their clinical efficacy and delivery protocols differ significantly. DBS is invasive and is often given in extremely late-stage PD when L-dopa treatments are no longer effective. Unfortunately, at this stage, we acknowledge that it is unlikely that any intervention is capable of reversing serious pathology. While tDCS and rTMS could be applied earlier in disease onset, they typically only target cortical regions due to the electrode placement. In contrast, ECT targets can be more widespread, with greater options for targeting various deep brain structures related to PD. Hence, we propose a mechanism by which neuromodulatory treatments, specifically ECT given early in PD pathology, may be acting on CD4 + and CD8 + T cells through the activation of their VGCC. We will speak generally to neuromodulation since all treatments are capable of activating VGCC on T cells, though we highlight that application of ECT early in disease progression will likely have the best immunomodulatory effects.

## 6 Proposed mechanisms whereby neuromodulation can affect α-syn accumulation

Since the accumulation of α-syn fibrils is linked to neuronal loss and motor symptoms, these fibrils pose an interesting target for PD treatment. We have recently shown that direct current stimulation (DCS) in SH-SY5Y dopaminergic cells results in decreases in intracellular α-syn aggregation and accumulation and this appeared to stem from excretion of the fibrils from the cytosol into the extracellular media (Ross et al., 2020). However, α-syn is easily able to re-enter the cells and cause damage unless it can be degraded. This, we believe, is where T cells come into play. Presently, we propose three separate possible, albeit similar mechanisms, whereby neuromodulation may ultimately serve to clear α-syn accumulation: (1) Neuromodulation can activate neurons, causing them to release misfolded α-syn, allowing for T cell recognition and subsequent degradation in the extracellular milieu, (2) Neuromodulation can activate CD4 + T cells through VGCC, and (3) Neuromodulation can cause conformational changes to α-syn that allow for T cell antigenic recognition and degradation.

### 6.1 Neuromodulatory treatments can activate neurons, causing them to eject misfolded α-syn, allowing for T cell recognition and clearance

We previously demonstrated, using an *in vitro* preparation, that electrical stimulation can cause cells to excrete α-syn pre-formed fibrils (Ross et al., 2020). Other studies have likewise shown that neuronal depolarization can increase the motility of physiological α-syn monomers. Specifically, increased neuronal activity promoted dissociation from pre-synaptic terminals and Ca^2+^ dependant excretion of α-syn into extracellular space (Emmanouilidou et al., 2010; Emmanouilidou et al., 2016; Fortin et al., 2005). These studies suggest an interplay between Ca^2+^ levels and α-syn clearance that is hypothesized to involve heat shock protein 70 (HSP70) induced activation of autophagy (Gao et al., 2019). Autophagy, in this case, can promote α-syn clearance following secretion (Li et al., 2006). Of course, autophagy can also regulate CD4 + T cell homeostasis among other things, raising the possible involvement of these cells in α-syn clearance following secretion (Sulzer et al., 2017; Lindestam Arlehamn et al., 2020).

Our working hypothesis is that neuromodulatory treatments promote toxic α-syn fibril clearance in a Ca^2+^ -dependent fashion, together with CD4 + T cell recognition of these fibrils. As already mentioned (Sulzer et al., 2017), CD4 + T cells can recognize aberrant forms of the α-syn protein through specific antigenic sequences. However, their recognition is likely limited to the extracellular space, which means initial α-syn secretion first is critical. In contrast, if α-syn fibrils are not secreted, then CD8 + T cells could potentially induce attacks directly on a-syn bearing neurons resulting in their death (Galiano-Landeira et al., 2020). This is assuming that the a-syn “infected” neurons express some signature or marker that is recognized by the CD8 + T cells. This conceptual model is tangentially supported by studies showing high levels of CD8 + T cells in the substantia nigra of PD patients (Subbarayan et al., 2020; Kurkowska-Jastrzebska et al., 1999a; Hisanaga et al., 2001). It is conceivable that ECT could induce α-syn secretion, thereby preventing CD8 + induced cell death and instead invoking beneficial CD4 + mediated extracellular α-syn clearance.

### 6.2 Neuromodulatory treatments can activate CD4 + T cells through VGCC

The concentration of ions distributed intra- and extra-cellularly directly impacts the electrical activity of any cell that possesses ion channels. Consequently, the electrical impulses delivered from DBS, ECT and tDCS can alter the ionic concentration of neurons, resulting in depolarization and neuronal firing. It is reasonable to assume that the electrical impulses from such neuromodulatory treatments can also affect T cells through their VGCCs ion channels. Several studies have in fact, demonstrated that CD4 + T cells express high levels of the Ca_*v*_1.1-1.4 pore-forming subunit family (Badou et al., 2013; Badou et al., 2006; Bannister and Beam, 2013). The expression of Ca_*v*_1.1 increases as CD4 + T cells become primed by antigenic exposure, suggesting that these channels play an important role in antigen-specific responses to pathogens (Badou et al., 2006). The influx of calcium through L-type calcium channels in CD4 + T cells activates protein kinase C-dependent processes that modulate Ca_*v*_1.2 channels and are critical for the ability of these cells to facilitate humoral immunity (Badou et al., 1997).

Although some data suggest ECT may result in an overall reduction of T lymphocyte counts (van Buel et al., 2015), others found increases in the number of activated T cells upon completion of ECT (Fischler et al., 1992). These results are not necessarily conflicting as it is possible that neuromodulatory treatments which activate T cells, do so differently depending upon the particular state of the T cell at the time of stimulation. More specifically, T cells that already have recognized a specific epitope, would be expected to show a very different proliferative state then those that are antigen naïve.

**FIGURE 3:**
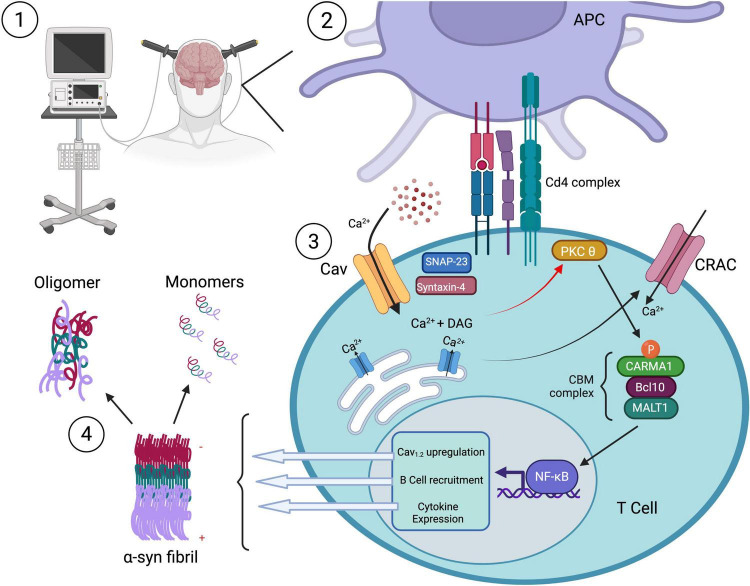
The effect of neuromodulation on CD4 + mediated immune function. (1) Neuromodulatory treatment (e.g., ECT) stimulates Ca^2+^ influx through L-type Cav channels on CD4^+^ T cells, triggering (3) intracellular signaling cascades. (2) Antigen-presenting cell (APC) engagement via the CD4 complex activates PKCθ, leading to recruitment of the CBM complex (CARMA1, Bcl10, MALT1) and downstream NF-κB–mediated transcription. This results in Cav1.2 channel upregulation, B cell recruitment, and cytokine expression, amplifying the adaptive immune response. Activated CD4^+^ T cells drive B cell activation and antibody production, potentially enabling antibody-dependent clearance of extracellular aggregated α-synuclein species (4). T cell responses to α-synuclein may help promote immune-mediated removal of toxic fibrils from the PD brain. Created with BioRender.com.

As previously mentioned, CD4 + T cells are responsible for directing the humoral branch of adaptive immunity through the stimulation of B cells and secretion of antibodies. Such activated B cell-derived antibodies could act to potentially neutralize any extracellular aggregated α-syn protein. If ECT causes calcium influx through L-type calcium channels on CD4 + T cells, then subsequent activation of B cells could conceivably prevent α-syn accumulation through antibody-dependent means (Figure 3). Furthermore, CD4 + CD25 + Treg cells have displayed neuroprotective effects in an MPTP model of PD, resulting in greater than 90% protection of the nigrostriatal system (Reynolds et al., 2007). In this model, the authors hypothesize that activated Tregs can alter the microglia phenotype from a toxic to a more trophic “protective” state resulting in neuronal survival (Reynolds et al., 2007). It is possible that neuromodulatory treatments also result in an activation of CD4 + Treg cells in a calcium-dependant manner. Since Tregs have been shown to suppress microglia ROS production (Reynolds et al., 2007), activation of these CD4 + Treg cells may improve motor function by restraining microglia-mediated cytotoxicity directed at pathological α-syn in dopaminergic cells (Table 2).

**TABLE 2:** Mechanisms by which T cell activation via neuromodulatory treatments may be exerting its beneficial effects on PD motor symptoms.

T cell activation results in	Causing	Evidence	References
Ca_v_ upregulation	Stronger response to known antigen (α-syn)	(1) Ca_V_1.4 is essential for modulation of intracellular Ca^2+^ stores and T cell receptor (TCR)-induced rises in cytosolic-free Ca^2+^ (2) Ca_v_1.4 null T cells exhibit defective calcium homeostasis and decreased survival (3) CD4 + T cells lacking Ca_v_ regulatory subunits β3 and β4 show impaired Ca^2+^ responses to T cell antigen receptor stimulation	(Omilusik et al., 2011) (Pelletier and Savignac, 2013) (Badou et al., 2006)
B cell recruitment	Antibody-dependant clearance of α-syn	(1) IgG^+^ memory B cells from PD patients have α-syn reactivity, inhibiting α-syn seeding (2) B cell numbers are depleted in α-syn knockout mice	(1) (Li et al., 2019) (2) (Xiao et al., 2014)
Cytokine expression	Mediate inflammation	(Figure 1)	(Zhu et al., 2010) (Luckheeram et al., 2012)
Increased activation of CD4 + Treg cells	Dopaminergic neuronal survival	(1) CD4 + CD25 + Treg cells have neuroprotective effects in an MPTP model of PD, resulting in greater than 90% protection of the nigrostriatal system. (2) α-syn- specific Tregs protect against motor function deficits in MPTP mouse model of PD	(1) (Reynolds et al., 2007) (2) (Park and Min, 2020)

### 6.3 Neuromodulatory treatment causes conformational changes to α-syn

It is currently unknown whether it is possible to induce the reversal of α-syn fibrils back to a monomer structural form. It is however known that direct electric current application can generally cause conformation changes in proteins (Calzia et al., 2009) and can also cause consistent inactivation of phospholipases (Calzia et al., 2009; Broersen et al., 2018). These findings suggest that current-inducing neuromodulatory treatments may have the potential to influence the structural characteristics of the α-syn protein. Furthermore, it is not outside the realm of possibility that such treatments might be able to revert the fibrilization of the protein by interfering with the hydrophobic bonds of the secondary helical structures.

**FIGURE 4:**
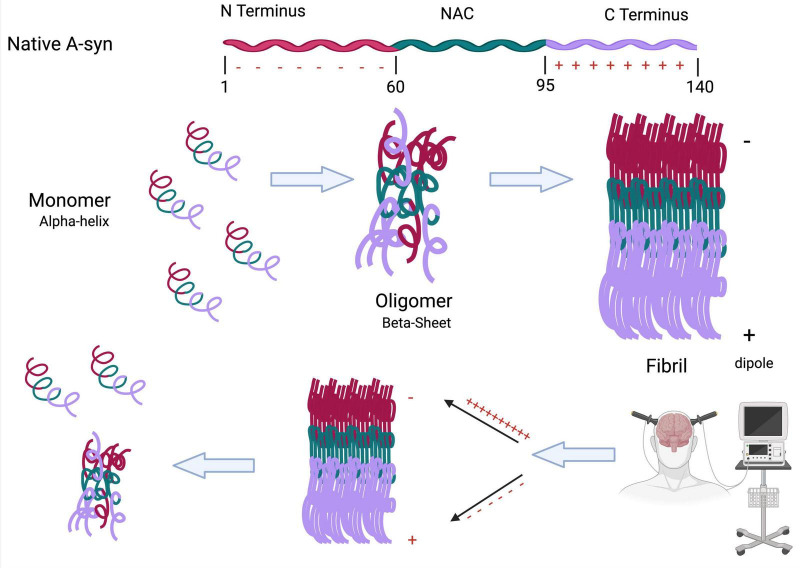
Neuromodulation can cause conformational changes to toxic α-syn fibrils. The folding of α-syn from a monomer to a fibril is highly dependent on the positively charged C terminus and negatively charged N terminus. This charge separation creates a molecular dipole that stabilizes the fibrillar form. We propose that neuromodulatory treatments, such as ECT, may disrupt this dipole alignment, leading to structural destabilization of the fibril. This could promote reversion to earlier, less toxic conformations (monomeric or oligomeric), potentially exposing epitopes that enhance T cell recognition and immune-mediated clearance (Created with BioRender.com).

While it is highly probable that T cells can detect α-syn, it is not clear which form of the protein, nor the exact antigens that might be detected. However, the absence of α-syn-specific T cell responses in the healthy brain (Gemechu and Bentivoglio, 2012) suggests that T cells may recognize aberrant forms of the protein. One recent study reported that CD4 + T cell responses against α-syn epitopes can be found up to 10 years before the onset of PD symptoms, with the highest levels occurring shortly following motor diagnosis, followed by a sharp decline thereafter (Lindestam Arlehamn et al., 2020). The sharp decline in CD4 + T cell levels as the disease progresses may be due to increased folding of α-syn fibrils into more aggregated forms, which could block recognition epitopes. It is theoretically possible that neuromodulatory treatments may be able to alter the structure of α-syn aggregates to expose epitopes and enhance T-cell recognition (Figure 4).

## 7 Conclusion and future considerations

Parkinson’s disease (PD) is increasingly recognized as a multifaceted disorder involving not only dopaminergic dysfunction but also significant neuroimmune alterations. Accumulating evidence points to a critical role for adaptive immunity, with CD4 + T cells recognizing α-synuclein (α-syn) epitopes and contributing to the inflammatory response. In parallel, neuromodulatory treatments such as ECT and TMS—which are already in clinical use for other brain disorders—are gaining interest for their potential to improve PD symptoms by mechanisms that may extend beyond neuronal circuitry, possibly involving immune modulation through voltage-gated calcium channels (VGCCs) (Popeo and Kellner, 2009; Grover et al., 2018; Fall et al., 1995; Usui et al., 2011; Berg, 2011). However, most neuromodulatory treatments are delivered during the later stages of the disease and the research has generally been limited to how these treatments enhance neural circuit function in the basal ganglia. With data emerging that PD is also linked to immune responses, it is plausible that neuromodulatory treatments may also exert beneficial effects in part through immunomodulation, which may be mediated by VGCCs.

Neuromodulation can activate VGCC and promote Ca^2+^ influx, leading to increased cytokine production and transcriptional changes that promote humoral immune responses. The ability of CD4 + T cells to recognize α-syn epitopes suggests that they could neutralize α-syn aggregation and spread. Yet, we also recognize the negative impact that excessive immune activation plays in disease progression. For example, chronic or excessive immune activation has been shown to exacerbate neuroinflammation and dopaminergic neurodegeneration in PD models (Reynolds et al., 2007; Harms et al., 2013; Xu et al., 2023). In particular, prolonged activation of microglia and infiltration of pro-inflammatory T cells have been associated with worsened neuronal death and disease progression (Brochard et al., 2008). Thus, a better understanding of the complex interplay and balance between pro and anti-inflammatory factors will require additional research to determine optimal modulatory treatments.

Preclinical work in modulation of T cell responses suggests neuroprotective effects are highly dependent on the timing of delivery (Brochard et al., 2009; Reynolds et al., 2007). While highly speculative, it may be that supporting immune activation through targeted neuromodulatory treatment at a prodromal stage of PD could alter the disease course. To address these concerns, future *in vivo* studies should employ time- and dose- controlled neuromodulatory treatments in α-syn models to assess how different activation windows and intensities effect neuronal survival and motor function. Similarly, *in vitro* studies may wish to clarify the conditions in which immune activation shifts from beneficial to detrimental. These approaches will help to identify the therapeutic window where neuromodulatory treatments can protect against PD pathology without exacerbating symptoms.
